# 1-Chloro-4-[2-(4-chloro­phen­yl)eth­yl]benzene and its bromo analogue: crystal structure, Hirshfeld surface analysis and computational chemistry

**DOI:** 10.1107/S2056989019004742

**Published:** 2019-04-12

**Authors:** Mukesh M. Jotani, See Mun Lee, Kong Mun Lo, Edward R. T. Tiekink

**Affiliations:** a Department of Physics, Bhavan’s Sheth R. A. College of Science, Ahmedabad, Gujarat 380001, India; b Research Centre for Crystalline Materials, School of Science and Technology, Sunway University, 47500 Bandar Sunway, Selangor Darul Ehsan, Malaysia

**Keywords:** crystal structure, 1,2-bis­(phen­yl)ethane, Hirshfeld surface analysis, inter­action energies

## Abstract

The crystal and mol­ecular structures of C_14_H_12_Cl_2_, (I), and C_14_H_12_Br_2_, (II), are described. The asymmetric unit of (I) comprises two independent mol­ecules, *A* and *B*, each disposed about a centre of inversion. Each mol­ecule approximates mirror symmetry [the C_b_—C_b_—C_e_—C_e_ torsion angles = −83.46 (19) and 95.17 (17)° for *A*, and −83.7 (2) and 94.75 (19)° for *B*; b = benzene and e = ethyl­ene]. By contrast, the mol­ecule in (II) is twisted, as seen in the dihedral angle of 59.29 (11)° between the benzene rings *cf*. 0° in (I). The mol­ecular packing of (I) features benzene-C—H⋯π(benzene) and Cl⋯Cl contacts that lead to an open three-dimensional (3D) architecture that enables twofold 3D–3D inter­penetration. The presence of benzene-C—H⋯π(benzene) and Br⋯Br contacts in the crystal of (II) consolidate the 3D architecture. The analysis of the calculated Hirshfeld surfaces confirm the influence of the benzene-C—H⋯π(benzene) and *X*⋯*X* contacts on the mol­ecular packing and show that, to a first approximation, H⋯H, C⋯H/H⋯C and C⋯*X*/*X*⋯C contacts dominate the packing, each contributing about 30% to the overall surface in each of (I) and (II). The analysis also clearly differentiates between the *A* and *B* mol­ecules of (I).

## Abstract 2

Two independent mol­ecules comprise the asymmetric unit of the chloro compound, each disposed about a centre of inversion. Each mol­ecule approximates mirror symmetry. By contrast, the bromo compound is significantly twisted [dihedral angle between the benzene rings = 59.29 (11)° *cf*. 0° for the chloro-containing mol­ecules].

## Chemical context   

The synthesis and physical characterization of the title compound, 1-chloro-4-[2-(4-chloro­phen­yl)eth­yl]benzene, C_14_H_12_Cl_2_, (I), has been reported by several research groups over the years (Otsubo *et al.*, 1980 ▸; Bestiuc *et al.*, 1985 ▸; Parnes *et al.*, 1989 ▸; Hu *et al.*, 2011 ▸; Liu & Li, 2007 ▸). In the same way, the bromo analogue of (I), 1-bromo-4-[2-(4-bromo­phen­yl)eth­yl]benzene, C_14_H_12_Br_2_, (II), has been described previously (Golden, 1961 ▸; Otsubo *et al.*, 1980 ▸; Remizov *et al.*, 2005 ▸; Liu & Li, 2007 ▸). Despite this inter­est, crystallographic characterization is lacking. Recently, compounds (I) and (II) became available as minor side-products during the synthesis of the respective tri(4-halobenz­yl)tin hydroxide from the reaction of tri(4-halobenz­yl)tin halide and sodium hydroxide. Herein, the crystal and mol­ecular structures of (I) and (II) are described. The structures are not isostructural and in order to gain further insight into the mol­ecular packing, the structures were subjected to an analysis of their Hirshfeld surfaces along with some computational chemistry.
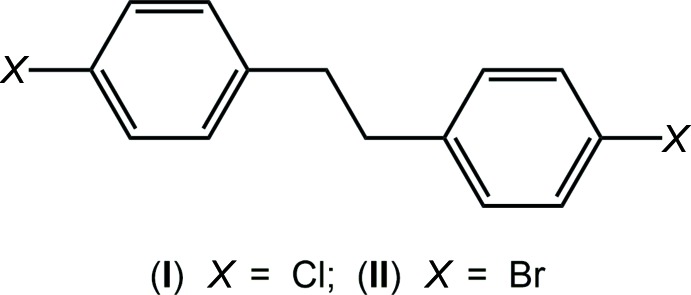


## Structural commentary   

**Figure 1:**
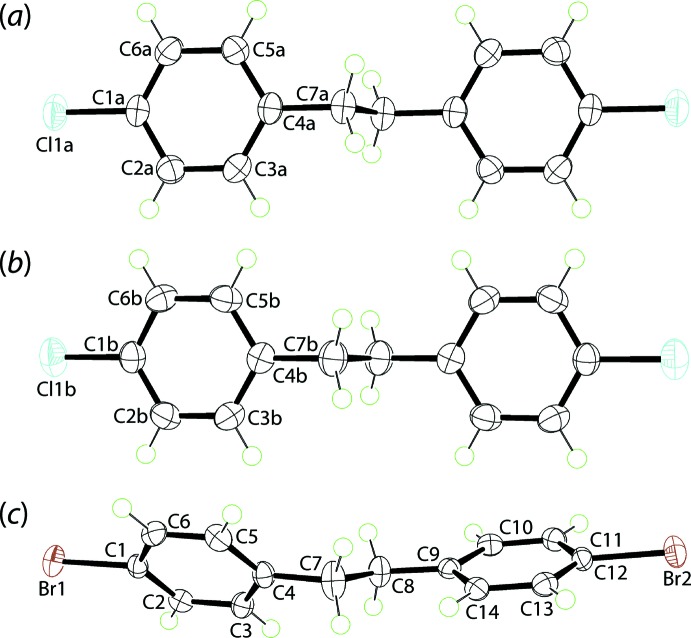
The mol­ecular structures of (*a*) the Cl1*A*-containing mol­ecule of (I), (*b*) the Cl1*B*-containing mol­ecule of (I) and (*c*) the mol­ecule of (II) showing the atom-labelling scheme and displacement ellipsoids at the 70% probability level. Unlabelled atoms in (*a*) and (*b*) are related by the symmetry operations 1 − *x*, 1 − *y*, 1 − *z* and 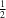 − *x*, 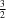 − *y*, 1 − *z*, respectively.

The two independent mol­ecules comprising the asymmetric unit of (I) are shown in Fig. 1 ▸(*a*) and (*b*); each is disposed about a centre of inversion. The mol­ecules present very similar features and, from inversion symmetry, comprise parallel benzene rings. The C3*A*—C4*A*—C7*A*—C7*A* ^i^ and C5*A*—C4*A*—C7*A*—C7*A* ^i^ torsion angles of −83.46 (19) and 95.17 (17)° highlight deviations from mirror symmetry in the mol­ecule [symmetry operation: (i) 1 − *x*, 1 − *y*, 1 − *z*]. These values are equal within experimental error and are very close to the equivalent angles for the second independent mol­ecule of −83.7 (2) and 94.75 (19)°, respectively [symmetry operation: (ii) 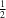 − *x*, 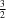 − *y*, 1 − *z*].

The mol­ecule of (II) is shown in Fig. 1 ▸(*c*) and does not feature the mol­ecular symmetry of (I). The difference in the conformation in (II), *cf*. (I), is seen immediately in the magnitude of the dihedral angle formed between the benzene rings of 59.29 (11)°, indicating an inclined disposition. The central torsion angle, *i.e*. C4—C7—C8—C9 of 172.1 (2)°, deviates from the 180° angles observed for the two independent mol­ecules in (I). The twist in the mol­ecule of (II) is reflected in the four torsion angles C3—C4—C7—C8 [46.6 (3)°], C5—C4—C7—C8 [−134.8 (2)°], C7—C8—C9—C14 [16.4 (3)°] and C7—C8—C9—C10 [−163.7 (2)°].

**Figure 2:**
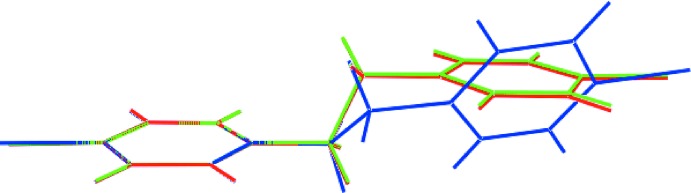
Overlap diagram of the (*a*) Cl1*A*-mol­ecule in (I) (red image), (*b*) Cl1*B*-mol­ecule in (I) (green) and (*c*) the mol­ecule in (II) (blue). Mol­ecules have been overlapped so that the C1-benzene rings are coincident.

The conformational differences between the mol­ecules in (I) and (II) are highlighted in the overlay diagram shown in Fig. 2 ▸.

## Supra­molecular features   

**Figure 3:**
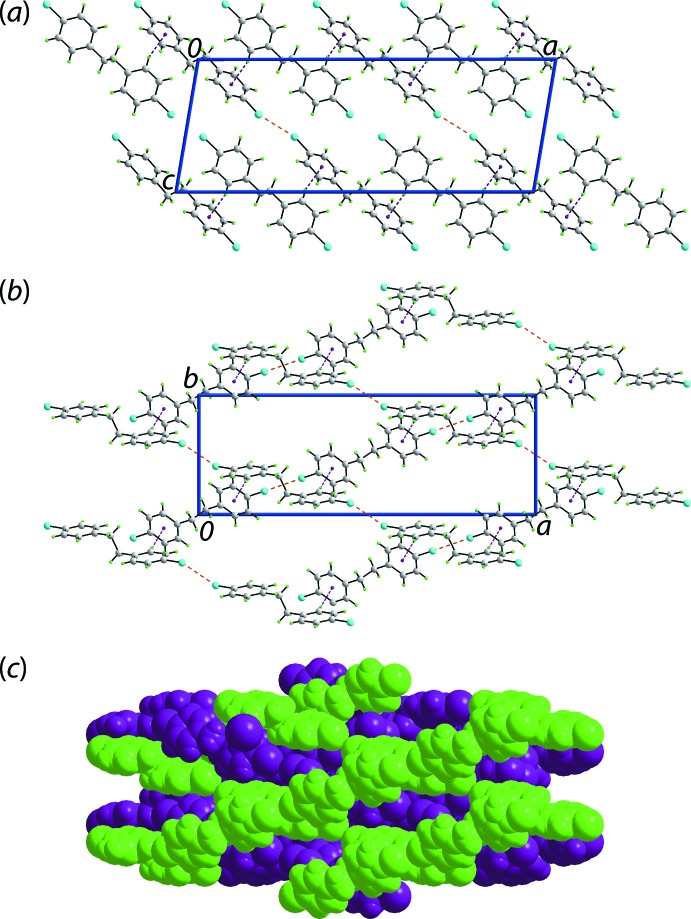
Mol­ecular packing in (I): (*a*) a view of the supra­molecular layer parallel to [1
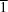
0] sustained by C—H⋯π and Cl⋯Cl contacts shown as purple and orange dashed lines, respectively, (*b*) a view of half of the unit-cell contents shown in projection down the *c* axis and (*c*) an image highlighting the twofold inter­penetration in space-filling mode.

In the crystal of (I), the main point of contact between the independent mol­ecules comprising the asymmetric unit are of the type benzene-C—H⋯π(benzene), Table 1 ▸. The result is the formation of a supra­molecular chain along the *a*-axis direction. Chains are connected into a supra­molecular layer *via* end-on Cl1*A*⋯Cl1*A* ^iii^ contacts [3.3184 (7) Å and C1—C11*A⋯*Cl1*A* ^iii^ = 164.61 (5)° for symmetry operation (iii) 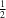 − *x*, 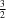 − *y*, −*z*], Fig. 3 ▸(*a*). The topology of the layer is flat and connections between the layers that stack along [1
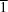
0] are weaker end-on Cl1*B*⋯Cl1*B* ^iv^ contacts [3.4322 (7) Å and C1*B*—Cl1*B*⋯Cl1*B* ^iv^ = 155.19 (5)° for symmetry operation (iv) 1 − *x*, 2 − *y*, 2 − *z*], which lead to a three-dimensional (3-D) architecture. As seen from Fig, 3(*b*), there are large voids defined by the aforementioned contacts which enables twofold, 3D–3D inter­penetration, Fig. 3 ▸(*c*).

**Figure 4:**
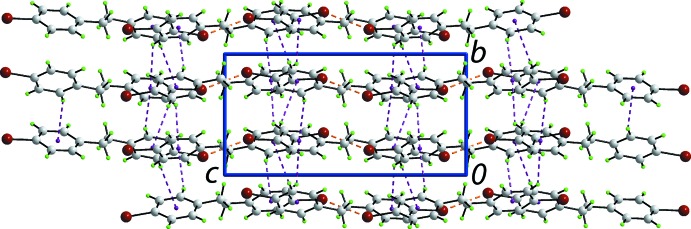
Mol­ecular packing in (II): a view of the unit-cell contents shown in projection down the *a* axis, highlighting C—H⋯π and Br⋯Br contacts as purple and orange dashed lines, respectively.

The 3-D architecture of (II) is supported by benzene-C—H⋯π(benzene) and Br⋯Br contacts. Globally, mol­ecules assemble in the *ac* plane and are connected to layers along [010] by benzene-C—H⋯π(benzene) contacts, Table 2 ▸. Further, lateral inter­actions are Br1⋯Br2^i^ [3.5242 (4) Å, C1—Br⋯Br2^i^ = 144.67 (7)° and C12^i^—Br2^i^⋯Br1 = 154.39 (7)° for symmetry operation (i) 1 + *x*, *y*, 1 + *z*; Fig. 4 ▸].

## Hirshfeld surface analysis   

The Hirshfeld surface calculations for (I) and (II) were performed in accord with established procedures (Tan *et al.*, 2019 ▸) with the aid of *Crystal Explorer* (Turner *et al.*, 2017 ▸) to determine the influence of weak inter­molecular inter­actions upon the mol­ecular packing in the absence of conventional hydrogen bonds.

**Figure 5:**
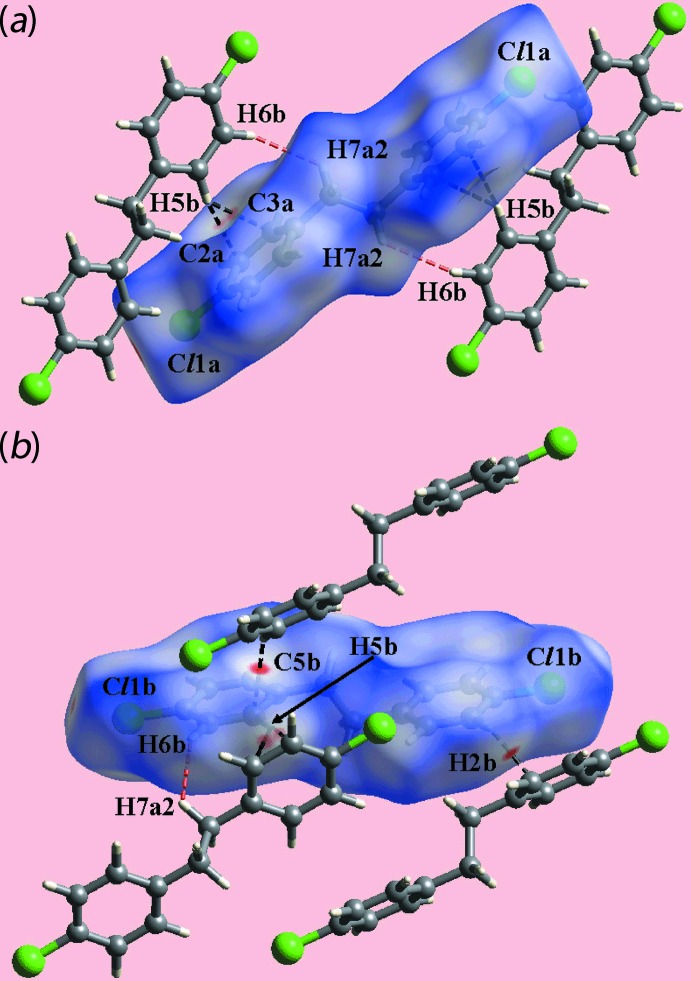
Views of the Hirshfeld surfaces for (I) mapped over *d*_norm_ for (*a*) mol­ecule *A* [in the range −0.103 to +1.259 arbitrary units] and (*b*) mol­ecule *B* [−0.072 to +1.234 arbitrary units] highlighting the short inter­atomic C⋯H/H⋯C and H⋯H contacts through black and red dashed lines, respectively.

In the crystal of (I), with two independent mol­ecules, labelled *A* and *B*, disposed about a centre of inversion the presence of faint-red spots near the benzene-C2*A*, C3*A* and H5*B* atoms in the images of Hirshfeld surfaces mapped over *d*_norm_ in Fig. 5 ▸ represent C—H⋯π contacts, Tables 1 ▸ and 3 ▸. The diminutive red spot viewed near the benzene-C5*B* atom in Fig. 5 ▸(*b*) indicates the effect of a short inter­atomic C5*B*⋯H2*B* contact, Table 3 ▸. Also, the presence of diminutive red spots near the terminal chlorine atoms of both independent mol­ecules in Fig. 5 ▸ are due to the formation of short inter­atomic Cl⋯Cl contacts, Table 3 ▸.

**Figure 6:**
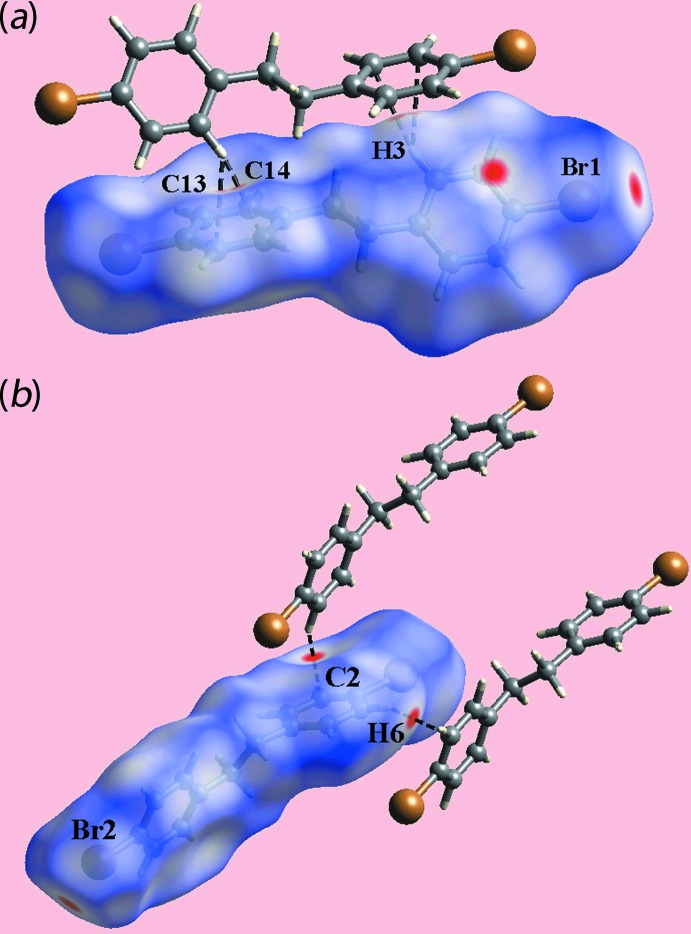
Views of the Hirshfeld surfaces for (II) mapped over *d*_norm_ [in the range −0.104 to +1.172 arbitrary units] highlighting the short inter­atomic C⋯H/H⋯C contacts through black dashed lines.

In the crystal of (II), the bright-red spots near the bromine atoms on the Hirshfeld surfaces mapped over *d*_norm_ in Fig. 6 ▸ indicate inter­atomic Br⋯Br contacts, Table 3 ▸, whereas those near the benzene-C2 and H6 atoms in Fig. 6 ▸(*b*) indicate short inter­atomic C—H⋯π inter­actions, Table 3 ▸. The presence of faint-red spots near the benzene-C13, C14 and H3 atoms in Fig. 6 ▸(*a*) also reflect the presence of C—H⋯π contacts, Table 3 ▸.

**Figure 7:**
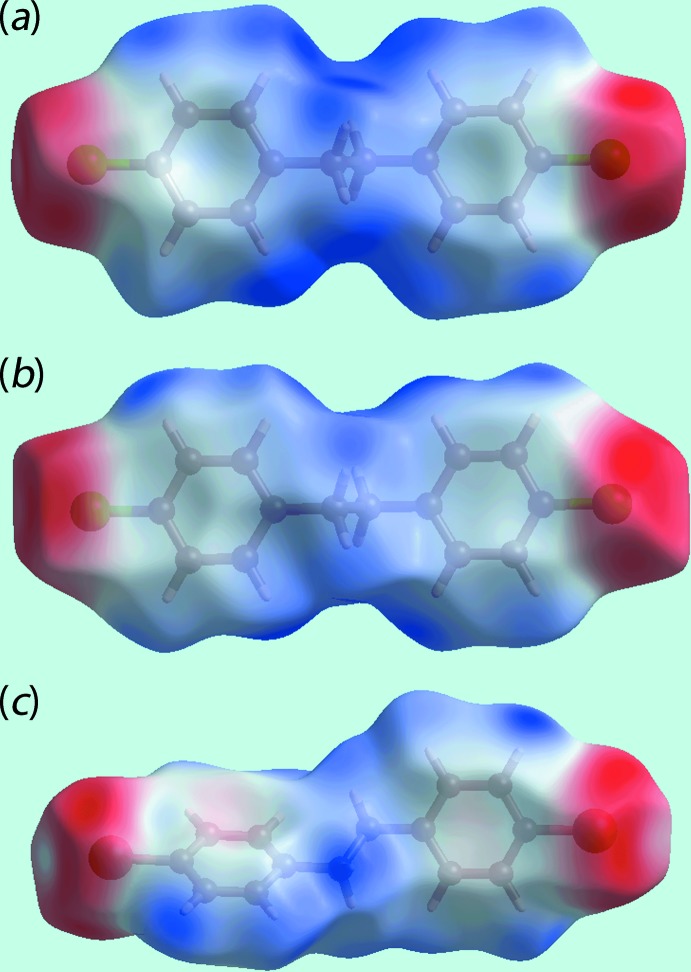
Views of the Hirshfeld surfaces mapped over the calculated electrostatic potential for (*a*) (I), mol­ecule *A* [−0.032 to +0.035 a.u.], (*b*) (I), mol­ecule *B* in [−0.033 to +0.044 a.u.] range and (*c*) (II) [−0.022 to +0.039 a.u.]. The red and blue regions represent negative and positive electrostatic potentials, respectively.

**Figure 8:**
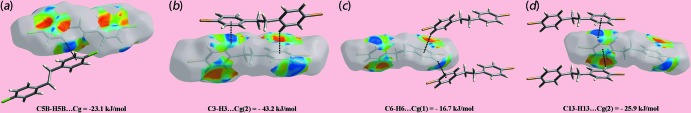
Views of the Hirshfeld surfaces mapped with the shape index property for (*a*) (I), mol­ecule *B*, (*b*)–(*d*) (II), highlighting inter­molecular C—H⋯π inter­actions through black dotted lines.

From the views of Hirshfeld surfaces mapped over the calculated electrostatic potentials in Figs. 7 ▸(*a*) and (*b*) for the independent mol­ecules of (I) highlight the small deviations from putative mirror symmetry through the slight differences in the blue and red regions around the atoms of their surfaces corresponding, respectively, to positive and negative potentials. For (II), Fig.7(*c*), the donors and acceptors of the C—H⋯π inter­actions are viewed as blue bumps and light-red concave regions. Further, the donors and acceptors of the C—H⋯π contacts for each of (I) and (II) are also illustrated through black dotted lines on the Hirshfeld surfaces mapped with shape-index properties in Fig. 8 ▸.

**Figure 9:**
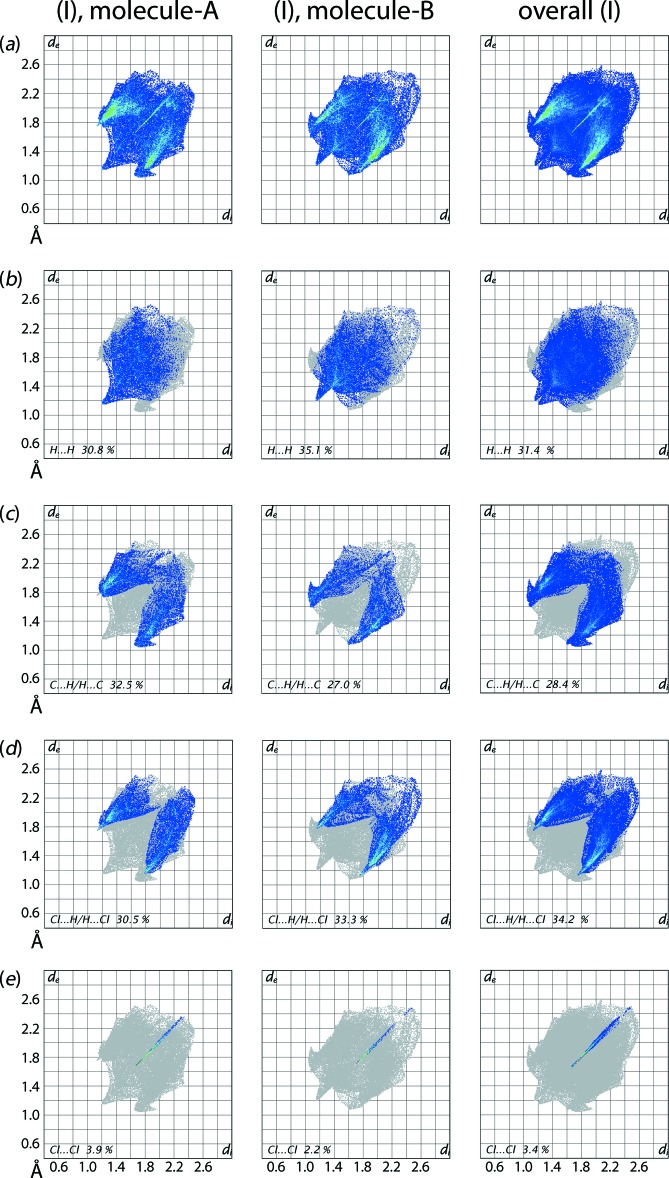
(*a*) The full two-dimensional fingerprint plot for mol­ecule *A* of (I), mol­ecule *B* of (I), and overall (I), and (*b*)–(*e*) those delineated into H⋯H, C⋯H/H⋯C, Cl⋯H/H⋯Cl and Cl⋯Cl contacts.

The overall two-dimensional fingerprint plot for the independent mol­ecules *A* and *B* as well as entire (I) are shown in Fig. 9 ▸(*a*), and those delineated into H⋯H, C⋯H/H⋯C, Cl⋯H/H⋯Cl and Cl⋯Cl contacts are illustrated in Fig. 9 ▸(*b*)–(*e*), respectively. The qu­anti­tative summary of percentage contributions from the different inter­atomic contacts to the respective Hirshfeld surfaces of *A*, *B* and (I) are presented in Table 4 ▸.

Some qualitative differences in the fingerprint plots are evident for mol­ecules *A* and *B*, confirming their distinct packing inter­actions. The complementary pair of forceps-like tips at *d*_e_ + *d*_i_ ∼2.3 Å in the fingerprint plots delineated into H⋯H contacts for *A* and *B* in Fig. 9 ▸(*b*) represent the short inter­atomic H⋯H contact, Table 3 ▸, which merge to form a pair of tips in the overall plot for (I). The fingerprint plots delineated into C⋯H/H⋯C contacts for mol­ecules *A* and *B* in Fig. 9 ▸(*c*) exhibit the clearest distinction between the inter­atomic contacts formed by the mol­ecules through the asymmetric distribution of points. The complementary distribution of points in the acceptor and donor regions of the plots for *A* and *B*, respectively, with the peaks at *d*_e_ + *d*_i_ ∼2.7 Å, are due to the formation of short inter­atomic C⋯H/H⋯C contacts between the benzene-C2*A*, C3*A* and H5*B* atoms, Table 3 ▸. Similar short inter­atomic contacts between benzene-C5*B* and H2*B* atoms of *B* results in forceps-like tips at *d*_e_ + *d*_i_ ∼2.7 Å in the acceptor region of the plot whereas it is merged within the tip of previously mentioned contact in the donor region. However, the respective plot for an overall structure is symmetric owing to the merging of the asymmetric distribution of points. The significant and quite similar contributions from Cl⋯H/H⋯Cl contacts to the Hirshfeld surfaces of *A*, *B* and overall (I), Fig. 9 ▸(*d*), have very little influence on the mol­ecular packing due to their inter­atomic distances being equal to or greater than the sum of their van der Waals radii. The linear distribution of points beginning from *d*_e_ + *d*_i_ ∼3.3 and 3.4 Å, Fig. 9 ▸(*e*), in the Cl⋯Cl delineated plots for *A* and *B*, respectively, indicate the presence of Cl⋯Cl inter­actions. The small contribution from C⋯C contacts to the Hirshfeld surface of (I) has a negligible effect on the packing.

**Figure 10:**
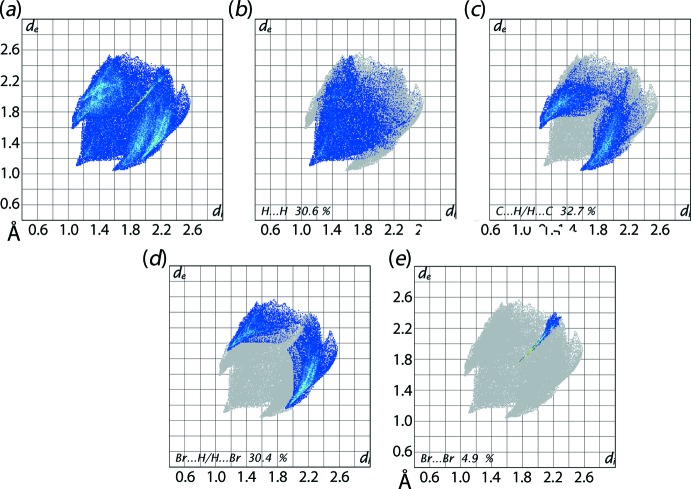
(*a*) The full two-dimensional fingerprint plot for (II), and (*b*)–(*e*) those delineated into H⋯H, C⋯H/H⋯C, Br⋯Br and Br⋯H/H⋯Br contacts.

Comparable fingerprint plots for (II) are shown in Fig. 10 ▸ and percentage contributions are collected in Table 4 ▸. The short inter­atomic H⋯H contact between symmetry-related ethyl­ene-H8*B* atoms is viewed as a single peak at *d*_e_ + *d*_i_ ∼2.2 Å in Fig. 10 ▸(*b*). In Fig. 10 ▸(*c*), delineated into C⋯H/H⋯C contacts, Table 3 ▸, the forceps-like tips at *d*_e_ + *d*_i_ ∼2.6 Å reflect the significant C—H⋯π contacts in the mol­ecular packing. The contribution of Br⋯H/H⋯Br contacts to the Hirshfeld surface of (II), Fig. 10 ▸(*d*), have very little influence on the packing due to their inter­atomic distances being around the sum of their van der Waals radii. The short inter­atomic Br⋯Br contacts in (II) are viewed as a thin, linear distribution of points initiating from *d*_e_ + *d*_i_ ∼3.5 Å, Fig. 10 ▸(*e*). As for (I), the small contribution from C⋯C contacts to the Hirshfeld surface of (II) has a negligible effect in the crystal.

## Computational chemistry   

The pairwise inter­action energies between the mol­ecules in the crystals of (I) and (II) were calculated by summing up four energy components, being electrostatic (*E*_ele_), polarization (*E*_pol_), dispersion (*E*_dis_) and exchange-repulsion (*E*_rep_) (Turner *et al.*, 2017 ▸). These energies were obtained by using the wave functions calculated at the B3LYP/6-31G(d,p) level theory for (I) and the HF/STO-3G level theory for (II). The individual energy components as well as total inter­action energy relative to reference mol­ecule within mol­ecular clusters out to 3.8 Å. The nature and strength of the energies for the key identified inter­molecular inter­actions are qu­anti­tatively summarized in Table 5 ▸. Dispersive components are dominant as conventional hydrogen bonding is not possible.

The significant contributions from the C—H⋯π inter­action and short inter­atomic C⋯H/H⋯C contacts in the crystal of (I) are evident from Table 5 ▸. Also notable, are the negligible energies associated with the Cl⋯Cl contacts due to the dominance of repulsive contributions. With respect to (II), it is evident from the comparison of the dispersive component as well as total energies for the different inter­actions that the strength of inter­actions in the crystal depend upon distance between the respective mol­ecules. The short Br⋯Br contacts in (II) also have very small inter­action energies.

**Figure 11:**
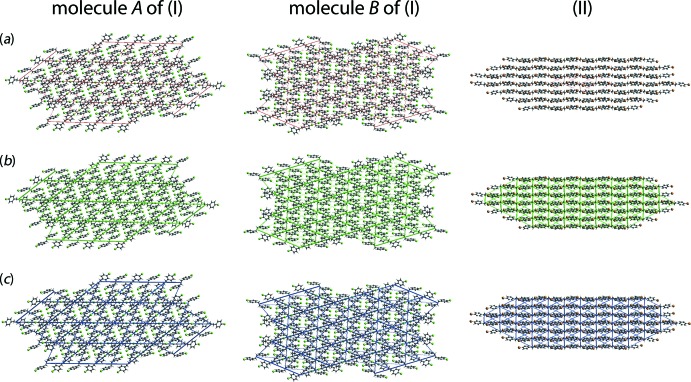
A comparison of the energy frameworks composed of (*a*) electrostatic potential force, (*b*) dispersion force and (*c*) total energy for cluster about a reference mol­ecule of *A* and *B* of (I), and for (II). The energy frameworks were adjusted to the same scale factor of 80 with a cut-off value of 2 kJ mol^−1^ within 4 × 4 × 4 unit cells.

The magnitudes of inter­molecular energies are represented graphically in the energy frameworks of Fig. 11 ▸. Here, the supra­molecular architecture of each crystal is viewed through the cylinders joining the centroids of mol­ecular pairs. The red (*E*_ele_), green (*E*_disp_) and blue (*E*_tot_) colour scheme represent the specified energy components. The radii of the cylinders are proportional to the magnitude of inter­action energies which are adjusted with a cut-off value of 2 kJ mol^−1^ within 4 × 4 × 4 unit cells. The energy frameworks constructed for the clusters about the independent mol­ecules *A* and *B* of (I) as well as that for (II) also indicate the distinct mode of supra­molecular association around the mol­ecules in the mol­ecular packing. The small effect of the electrostatic components and the significant influence of the dispersive components are clearly evident from the energy frameworks shown in Fig. 11 ▸.

## Database survey   

There are only four halo-substituted 1,2-bis­(phen­yl)ethyl­ene derivatives in the literature. The key structural parameters for these are summarized in Table 6 ▸. Only one literature structure is not disposed about a centre of inversion, namely the non-symmetric, mixed-halo structure (4-Br,2,6-F_2_C_6_H_2_)CH_2_CH_2_C_6_H_4_Br-4 (Galán *et al.*, 2016 ▸). Generally, the central C_e_—C_e_ (e = ethyl­ene) bonds are long in these compounds with the exception being the penta­bromo derivative, C_6_Br_5_CH_2_CH_2_C_6_Br_5_ (Köppen *et al.*, 2007 ▸).

## Synthesis and crystallization   

Tri(4-chloro­benz­yl)tin chloride was prepared by direct synthesis using tin powder (Merck) and 4-chloro­benzyl chloride (Sigma–Aldrich) in water (Sisido *et al.*, 1961 ▸). Tri(4-chloro­benz­yl)tin chloride (5.3 g, 10 mmol) was dissolved in 95% ethanol (150 ml) and to this was added dropwise 10% sodium hydroxide solution (4 ml). The resulting solution was heated for 1 h. After cooling, the white tri(4-chloro­benz­yl)tin hydroxide was filtered off and the filtrate was evaporated slowly to obtain a colourless crystalline solid which was identified crystallographically as (I). Yield: 0.28 g (0.11%). The bromo analogue was similarly obtained as a side-product from the base hydrolysis of tri(4-bromo­benz­yl)tin bromide. Tri(4-bromo­benzyl­tin) bromide was prepared from the reaction of tin powder (Sigma–Aldrich) and 4-bromo­benzyl bromide (Merck) in water (Sisido *et al.*, 1961 ▸). Tri(4-bromo­benz­yl)tin bromide (7.0 g, 10 mmol) was dissolved in 95% ethanol (150 ml) and to this was added 10% sodium hydroxide solution (4 ml). The resulting precipitation was heated for 1 h. After cooling, the yellow tri(4-bromo­benz­yl)tin hydroxide was filtered off and the filtrate was evaporated slowly to obtain a yellow crystalline solid which was identified crystallographically as (II). Yield: 0.25 g (0.07%)

## Refinement   

Crystal data, data collection and structure refinement details are summarized in Table 7 ▸. The carbon-bound H atoms were placed in calculated positions (C—H = 0.93–0.99 Å) and were included in the refinement in the riding-model approximation, with *U*_iso_(H) set to 1.2*U*_eq_(C). In the refinement of (II), owing to poor agreement the (111) reflection was omitted from the final cycles of refinement.

## Supplementary Material

Crystal structure: contains datablock(s) I, II, global. DOI: 10.1107/S2056989019004742/hb7814sup1.cif

Structure factors: contains datablock(s) I. DOI: 10.1107/S2056989019004742/hb7814Isup2.hkl

Supporting information file. DOI: 10.1107/S2056989019004742/hb7814Isup4.cml

Structure factors: contains datablock(s) II. DOI: 10.1107/S2056989019004742/hb7814IIsup3.hkl

Supporting information file. DOI: 10.1107/S2056989019004742/hb7814IIsup5.cml

CCDC references: 1908484, 1908483

Additional supporting information:  crystallographic information; 3D view; checkCIF report

## Figures and Tables

**Table 1:** Hydrogen-bond geometry (Å, °) for (I) *Cg*1 is the centroid of the (C1*A*–C6*A*) ring.

*D*—H⋯*A*	*D*—H	H⋯*A*	*D*⋯*A*	*D*—H⋯*A*
C5*B*—H5*B*⋯*Cg*1	0.93	2.62	3.4866 (15)	155

**Table 2:** Hydrogen-bond geometry (Å, °) for (II) *Cg*1 and *Cg*2 are the centroids of the (C1–C6) and (C9–C14) rings, respectively.

*D*—H⋯*A*	*D*—H	H⋯*A*	*D*⋯*A*	*D*—H⋯*A*
C3—H3⋯*Cg*2^i^	0.95	2.69	3.442 (2)	136
C6—H6⋯*Cg*1^ii^	0.95	2.91	3.704 (2)	141
C13—H13⋯*Cg*2^iii^	0.95	2.87	3.569 (2)	131

Symmetry codes: (i) 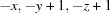; (ii) 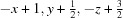; (iii) 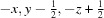.

**Table 3:** Summary of short inter­atomic contacts (Å) in (I)^*a*^

Contact	Distance	Symmetry operation
(I)		
H6*B*⋯H72*A*	2.35	*x*, *y*, *z*
H5*B*⋯C2*A*	2.75	*x*, *y*, *z*
H5*B*⋯C3*A*	2.72	*x*, *y*, *z*
H2*B*⋯C5*B*	2.67	*x*, 2 − *y*, 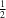 + *z*
C11*A*⋯Cl1*A*	3.3184 (7)	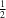 − *x*, 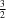 − *y*, −*z*
Cl1*B*⋯Cl1*B*	3.4322 (7)	1 − *x*, 2 − *y*, 2 − *z*
(II)		
H8*B*⋯H8*B*	2.21	−*x*, 2 − *y*, 1 − *z*
H3⋯C13	2.74	−*x*, 1 − *y*, 1 − *z*
H3⋯C14	2.72	−*x*, 1 − *y*, 1 − *z*
H6⋯C1	2.82	1 − *x*, 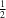 + *y*, 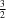 − *z*
H6⋯C2	2.62	1 − *x*, 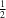 + *y*, 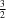 − *z*
H11⋯C6	2.80	−*x*, 2 − *y*, 1 − *z*
Br1⋯Br2	3.5242 (4)	1 + *x*, *y*, 1 + *z*

Notes: (*a*) The inter­atomic distances are calculated in *Crystal Explorer* (Turner *et al.*, 2017 ▸) whereby the *X*—H bond lengths are adjusted to their neutron values.

**Table 4:** Percentage contributions of inter­atomic contacts to the Hirshfeld surface for (I) and (II)

Contact	Percentage contribution			
	(I) - mol­ecule *A*	(I) - mol­ecule *B*	(I)	(II)
H⋯H	30.8	35.1	31.4	30.6
C⋯H/H⋯C	32.5	27.0	28.4	32.7
*X*⋯H/H⋯*X*	30.5	33.3	34.2	30.4
*X*⋯*X*	3.9	2.2	3.4	4.9
C⋯C	1.3	1.3	1.4	0.0
C⋯*X*/*X*⋯C	1.1	1.1	1.2	1.4

**Table 5:** Summary of inter­action energies (kJ mol^−1^) calculated for (I) and (II)

Contact	*E*_ele_	*E*_pol_	*E*_dis_	*E*_rep_	*E*_tot_
(I)					
Cl1*A*⋯Cl1*A*	−0.9	0.0	−3.3	7.6	0.9
Cl1*B*⋯Cl1*B*	−0.9	−0.1	−3.4	5.8	−0.4
C5—H5⋯*Cg*(C1*A*–C6*A*)	−8.5	−1.5	−33.5	26.1	−23.1
C5⋯H2*B*	−3.7	−0.8	−18.2	12.9	−12.3
(II)					
Br1⋯Br2	−2.2	−0.1	−4.9	8.4	0.1
C3—H3⋯*Cg*(C9–C14)	−14.6	−4.7	−62.4	38.3	−43.2
C6—H6⋯*Cg*(C1–C6)	−5.6	−1.5	−25.1	15.5	−16.7
C13—H13⋯*Cg*(C9–C14)	−8.9	−1.9	−30.9	14.2	−25.9
H11⋯C6	−5.0	−3.2	−50.6	24.7	−32.7
H8*B*⋯H8*B*	−5.0	−3.2	−50.6	24.7	−32.7

**Table 6:** Geometric data (Å, °) for halo-substituted 1,2-bis­(phen­yl)ethane structures

Ring 1	Ring 2	Symmetry	CH_2_—CH_2_	dihedral angle C_6_/C_6_	Reference
2-BrC_6_H_4_	2-BrC_6_H_4_	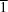	1.540 (7)	0	Kahr *et al.* (1995 ▸)
C_6_F_5_	C_6_F_5_	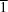	1.542 (3)	0	Krafczyk *et al.* (1997 ▸)
C_6_Br_5_	C_6_Br_5_	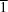	1.495 (13)	0	Köppen *et al.* (2007 ▸)
4-Br,2,6-F_2_C_6_H_2_	4-BrC_6_H_4_	–	1.522 (10)	1.67 (16)	Galán *et al.* (2016 ▸)
4-ClC_6_H_4_^*a*^	4-ClC_6_H_4_	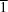	1.530 (2)	0	This work
		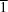	1.530 (3)	0	
4-BrC_6_H_4_	4-BrC_6_H_4_	–	1.516 (3)	59.29 (11)	This work

Notes: (*a*) Two independent mol­ecules comprise the asymmetric unit.

**Table 7:** Experimental details

	(I)	(II)
Crystal data		
Chemical formula	C_14_H_12_Cl_2_	C_14_H_12_Br_2_
*M*_r_	251.14	340.06
Crystal system, space group	Monoclinic, *C*2/*c*	Monoclinic, *P*2_1_/*c*
Temperature (K)	296	100
*a*, *b*, *c* (Å)	26.6755 (19), 9.3259 (7), 10.0405 (8)	10.8761 (2), 7.5157 (1), 15.6131 (3)
β (°)	99.560 (4)	106.177 (1)
*V* (Å^3^)	2463.1 (3)	1225.71 (4)
*Z*	8	4
Radiation type	Mo *K*α	Mo *K*α
μ (mm^−1^)	0.50	6.58
Crystal size (mm)	0.30 × 0.20 × 0.10	0.20 × 0.11 × 0.07

Data collection		
Diffractometer	Bruker SMART APEX CCD area detector	Bruker SMART APEX CCD area detector
Absorption correction	Multi-scan (*SADABS*; Sheldrick, 1996 ▸)	Multi-scan (*SADABS*; Sheldrick, 1996 ▸)
*T*_min_, *T*_max_	0.623, 0.746	0.546, 0.746
No. of measured, independent and observed [*I* > 2σ(*I*)] reflections	12061, 3090, 2627	11908, 3065, 2520
*R*_int_	0.026	0.035
(sin θ/λ)_max_ (Å^−1^)	0.669	0.669

Refinement		
*R*[*F* ^2^ > 2σ(*F* ^2^)], *wR*(*F* ^2^), *S*	0.033, 0.090, 1.04	0.026, 0.058, 1.03
No. of reflections	3090	3065
No. of parameters	145	145
H-atom treatment	H-atom parameters constrained	H-atom parameters constrained
Δρ_max_, Δρ_min_ (e Å^−3^)	0.31, −0.27	0.45, −0.41

Computer programs: *APEX2* (Bruker, 2008 ▸), *SAINT* (Bruker, 2008 ▸), *SHELXS97* (Sheldrick, 2008 ▸), *SHELXL2018/3* (Sheldrick, 2015 ▸), *ORTEP-3 for Windows* (Farrugia, 2012 ▸), *DIAMOND* (Brandenburg, 2006 ▸), *QMol* (Gans & Shalloway, 2001 ▸) and *publCIF* (Westrip, 2010 ▸).

## supplementary crystallographic information

### 1-Chloro-4-[2-(4-chlorophenyl)ethyl]benzene (I) Crystal data

**Table:**

C_14_H_12_Cl_2_	*F*(000) = 1040
*M**_r_* = 251.14	*D*_x_ = 1.354 Mg m^−^^3^
Monoclinic, *C*2/*c*	Mo *K*α radiation, λ = 0.71073 Å
*a* = 26.6755 (19) Å	Cell parameters from 4470 reflections
*b* = 9.3259 (7) Å	θ = 3.1–28.3°
*c* = 10.0405 (8) Å	µ = 0.50 mm^−^^1^
β = 99.560 (4)°	*T* = 296 K
*V* = 2463.1 (3) Å^3^	Prism, colourless
*Z* = 8	0.30 × 0.20 × 0.10 mm

### 1-Chloro-4-[2-(4-chlorophenyl)ethyl]benzene (I) Data collection

**Table:**

Bruker model? CCD area detector diffractometer	3090 independent reflections
Radiation source: fine-focus sealed tube	2627 reflections with *I* > 2σ(*I*)
Graphite monochromator	*R*_int_ = 0.026
φ and ω scans	θ_max_ = 28.4°, θ_min_ = 1.6°
Absorption correction: multi-scan (SADABS; Sheldrick, 1996)	*h* = −35→34
*T*_min_ = 0.623, *T*_max_ = 0.746	*k* = −11→12
12061 measured reflections	*l* = −13→13

### 1-Chloro-4-[2-(4-chlorophenyl)ethyl]benzene (I) Refinement

**Table:**

Refinement on *F*^2^	Primary atom site location: structure-invariant direct methods
Least-squares matrix: full	Hydrogen site location: inferred from neighbouring sites
*R*[*F*^2^ > 2σ(*F*^2^)] = 0.033	H-atom parameters constrained
*wR*(*F*^2^) = 0.090	*w* = 1/[σ^2^(*F*_o_^2^) + (0.046*P*)^2^ + 1.4907*P*] where *P* = (*F*_o_^2^ + 2*F*_c_^2^)/3
*S* = 1.04	(Δ/σ)_max_ = 0.001
3090 reflections	Δρ_max_ = 0.31 e Å^−^^3^
145 parameters	Δρ_min_ = −0.27 e Å^−^^3^
0 restraints	

### 1-Chloro-4-[2-(4-chlorophenyl)ethyl]benzene (I) Special details

**Table:**

Geometry. All esds (except the esd in the dihedral angle between two l.s. planes) are estimated using the full covariance matrix. The cell esds are taken into account individually in the estimation of esds in distances, angles and torsion angles; correlations between esds in cell parameters are only used when they are defined by crystal symmetry. An approximate (isotropic) treatment of cell esds is used for estimating esds involving l.s. planes.

### 1-Chloro-4-[2-(4-chlorophenyl)ethyl]benzene (I) Fractional atomic coordinates and isotropic or equivalent isotropic displacement parameters (Å^2^)

**Table:**

	*x*	*y*	*z*	*U*_iso_*/*U*_eq_	
Cl1A	0.30558 (2)	0.69571 (4)	0.08421 (3)	0.02990 (11)	
C1A	0.35616 (5)	0.64904 (15)	0.20984 (13)	0.0213 (3)	
C2A	0.39856 (5)	0.73741 (15)	0.23346 (13)	0.0227 (3)	
H2A	0.400392	0.819329	0.181667	0.027*	
C3A	0.43825 (5)	0.70121 (14)	0.33594 (13)	0.0224 (3)	
H3A	0.466935	0.759394	0.351988	0.027*	
C4A	0.43591 (5)	0.57952 (14)	0.41510 (13)	0.0202 (3)	
C5A	0.39306 (5)	0.49193 (14)	0.38683 (13)	0.0224 (3)	
H5A	0.391223	0.409246	0.437578	0.027*	
C6A	0.35314 (5)	0.52569 (15)	0.28454 (13)	0.0237 (3)	
H6A	0.324808	0.466334	0.266505	0.028*	
C7A	0.47884 (5)	0.54105 (15)	0.52636 (13)	0.0236 (3)	
H7A1	0.465754	0.482765	0.592893	0.028*	
H7A2	0.492801	0.628077	0.570743	0.028*	
Cl1B	0.44971 (2)	0.90465 (4)	0.91449 (4)	0.03360 (12)	
C1B	0.39561 (5)	0.87925 (14)	0.79317 (14)	0.0229 (3)	
C2B	0.34836 (6)	0.90808 (15)	0.82606 (14)	0.0271 (3)	
H2B	0.345284	0.938736	0.912478	0.033*	
C3B	0.30571 (5)	0.89060 (15)	0.72836 (15)	0.0274 (3)	
H3B	0.273843	0.910620	0.749858	0.033*	
C4B	0.30929 (5)	0.84387 (14)	0.59888 (13)	0.0223 (3)	
C5B	0.35742 (5)	0.81356 (15)	0.56992 (14)	0.0254 (3)	
H5B	0.360595	0.780451	0.484341	0.031*	
C6B	0.40064 (5)	0.83161 (15)	0.66564 (14)	0.0258 (3)	
H6B	0.432599	0.811998	0.644554	0.031*	
C7B	0.26239 (5)	0.82320 (15)	0.49401 (15)	0.0272 (3)	
H7B1	0.271471	0.832325	0.404772	0.033*	
H7B2	0.238029	0.898025	0.504017	0.033*	

### 1-Chloro-4-[2-(4-chlorophenyl)ethyl]benzene (I) Atomic displacement parameters (Å^2^)

**Table:**

	*U*^11^	*U*^22^	*U*^33^	*U*^12^	*U*^13^	*U*^23^
Cl1A	0.02150 (17)	0.0400 (2)	0.02582 (18)	0.00466 (13)	−0.00301 (12)	0.00464 (14)
C1A	0.0173 (6)	0.0266 (7)	0.0191 (6)	0.0037 (5)	0.0005 (5)	−0.0009 (5)
C2A	0.0226 (6)	0.0237 (6)	0.0222 (6)	0.0005 (5)	0.0051 (5)	0.0011 (5)
C3A	0.0186 (6)	0.0257 (6)	0.0230 (6)	−0.0014 (5)	0.0041 (5)	−0.0029 (5)
C4A	0.0175 (6)	0.0246 (6)	0.0186 (6)	0.0046 (5)	0.0031 (5)	−0.0031 (5)
C5A	0.0230 (6)	0.0206 (6)	0.0236 (6)	0.0016 (5)	0.0035 (5)	0.0009 (5)
C6A	0.0199 (6)	0.0247 (7)	0.0258 (7)	−0.0028 (5)	0.0017 (5)	−0.0024 (5)
C7A	0.0199 (6)	0.0292 (7)	0.0207 (6)	0.0045 (5)	0.0003 (5)	−0.0019 (5)
Cl1B	0.02872 (19)	0.0322 (2)	0.0356 (2)	−0.00182 (13)	−0.00707 (15)	−0.00290 (14)
C1B	0.0223 (6)	0.0199 (6)	0.0251 (6)	−0.0016 (5)	0.0001 (5)	0.0008 (5)
C2B	0.0296 (7)	0.0296 (7)	0.0233 (7)	−0.0023 (5)	0.0076 (6)	−0.0057 (5)
C3B	0.0214 (6)	0.0308 (7)	0.0313 (7)	−0.0006 (5)	0.0084 (5)	−0.0031 (6)
C4B	0.0211 (6)	0.0203 (6)	0.0250 (6)	−0.0005 (5)	0.0024 (5)	0.0028 (5)
C5B	0.0279 (7)	0.0278 (7)	0.0215 (6)	0.0031 (5)	0.0067 (5)	−0.0011 (5)
C6B	0.0216 (6)	0.0280 (7)	0.0286 (7)	0.0041 (5)	0.0069 (5)	0.0002 (5)
C7B	0.0251 (7)	0.0269 (7)	0.0276 (7)	−0.0002 (5)	−0.0011 (6)	0.0033 (6)

### 1-Chloro-4-[2-(4-chlorophenyl)ethyl]benzene (I) Geometric parameters (Å, º)

**Table:**

Cl1A—C1A	1.7424 (13)	Cl1B—C1B	1.7432 (13)
C1A—C6A	1.3829 (19)	C1B—C2B	1.381 (2)
C1A—C2A	1.3877 (18)	C1B—C6B	1.383 (2)
C2A—C3A	1.3899 (18)	C2B—C3B	1.383 (2)
C2A—H2A	0.9300	C2B—H2B	0.9300
C3A—C4A	1.3928 (19)	C3B—C4B	1.3895 (19)
C3A—H3A	0.9300	C3B—H3B	0.9300
C4A—C5A	1.3952 (18)	C4B—C5B	1.3917 (18)
C4A—C7A	1.5043 (17)	C4B—C7B	1.5079 (18)
C5A—C6A	1.3873 (18)	C5B—C6B	1.3832 (19)
C5A—H5A	0.9300	C5B—H5B	0.9300
C6A—H6A	0.9300	C6B—H6B	0.9300
C7A—C7A^i^	1.530 (2)	C7B—C7B^ii^	1.530 (3)
C7A—H7A1	0.9700	C7B—H7B1	0.9700
C7A—H7A2	0.9700	C7B—H7B2	0.9700
			
C6A—C1A—C2A	121.37 (12)	C2B—C1B—C6B	121.11 (12)
C6A—C1A—Cl1A	119.51 (10)	C2B—C1B—Cl1B	119.26 (11)
C2A—C1A—Cl1A	119.12 (10)	C6B—C1B—Cl1B	119.63 (10)
C1A—C2A—C3A	118.77 (12)	C3B—C2B—C1B	118.90 (13)
C1A—C2A—H2A	120.6	C3B—C2B—H2B	120.6
C3A—C2A—H2A	120.6	C1B—C2B—H2B	120.6
C2A—C3A—C4A	121.30 (12)	C2B—C3B—C4B	121.62 (13)
C2A—C3A—H3A	119.4	C2B—C3B—H3B	119.2
C4A—C3A—H3A	119.4	C4B—C3B—H3B	119.2
C3A—C4A—C5A	118.28 (12)	C3B—C4B—C5B	117.94 (12)
C3A—C4A—C7A	121.15 (12)	C3B—C4B—C7B	121.02 (12)
C5A—C4A—C7A	120.55 (12)	C5B—C4B—C7B	121.03 (12)
C6A—C5A—C4A	121.33 (12)	C6B—C5B—C4B	121.43 (13)
C6A—C5A—H5A	119.3	C6B—C5B—H5B	119.3
C4A—C5A—H5A	119.3	C4B—C5B—H5B	119.3
C5A—C6A—C1A	118.92 (12)	C5B—C6B—C1B	118.99 (12)
C5A—C6A—H6A	120.5	C5B—C6B—H6B	120.5
C1A—C6A—H6A	120.5	C1B—C6B—H6B	120.5
C4A—C7A—C7A^i^	112.14 (13)	C4B—C7B—C7B^ii^	112.23 (14)
C4A—C7A—H7A1	109.2	C4B—C7B—H7B1	109.2
C7A^i^—C7A—H7A1	109.2	C7B^ii^—C7B—H7B1	109.2
C4A—C7A—H7A2	109.2	C4B—C7B—H7B2	109.2
C7A^i^—C7A—H7A2	109.2	C7B^ii^—C7B—H7B2	109.2
H7A1—C7A—H7A2	107.9	H7B1—C7B—H7B2	107.9
			
C6A—C1A—C2A—C3A	−0.98 (19)	C6B—C1B—C2B—C3B	−1.0 (2)
Cl1A—C1A—C2A—C3A	178.52 (10)	Cl1B—C1B—C2B—C3B	178.62 (11)
C1A—C2A—C3A—C4A	−0.5 (2)	C1B—C2B—C3B—C4B	0.5 (2)
C2A—C3A—C4A—C5A	1.63 (19)	C2B—C3B—C4B—C5B	0.5 (2)
C2A—C3A—C4A—C7A	−179.71 (12)	C2B—C3B—C4B—C7B	179.08 (13)
C3A—C4A—C5A—C6A	−1.28 (19)	C3B—C4B—C5B—C6B	−1.2 (2)
C7A—C4A—C5A—C6A	−179.95 (12)	C7B—C4B—C5B—C6B	−179.72 (13)
C4A—C5A—C6A—C1A	−0.2 (2)	C4B—C5B—C6B—C1B	0.8 (2)
C2A—C1A—C6A—C5A	1.3 (2)	C2B—C1B—C6B—C5B	0.3 (2)
Cl1A—C1A—C6A—C5A	−178.18 (10)	Cl1B—C1B—C6B—C5B	−179.24 (11)
C3A—C4A—C7A—C7A^i^	−83.46 (19)	C3B—C4B—C7B—C7B^ii^	−83.7 (2)
C5A—C4A—C7A—C7A^i^	95.17 (17)	C5B—C4B—C7B—C7B^ii^	94.75 (19)

Symmetry codes: (i) −*x*+1, −*y*+1, −*z*+1; (ii) −*x*+1/2, −*y*+3/2, −*z*+1.

### 1-Chloro-4-[2-(4-chlorophenyl)ethyl]benzene (I) Hydrogen-bond geometry (Å, º)

*Cg*1 is the centroid of the (C1*A*–C6*A*) ring.

**Table:**

*D*—H···*A*	*D*—H	H···*A*	*D*···*A*	*D*—H···*A*
C5*B*—H5*B*···*Cg*1	0.93	2.62	3.4866 (15)	155

### 1-Bromo-4-[2-(4-chlorophenyl)ethyl]benzene (II) Crystal data

**Table:**

C_14_H_12_Br_2_	*F*(000) = 664
*M**_r_* = 340.06	*D*_x_ = 1.843 Mg m^−^^3^
Monoclinic, *P*2_1_/*c*	Mo *K*α radiation, λ = 0.71073 Å
*a* = 10.8761 (2) Å	Cell parameters from 3449 reflections
*b* = 7.5157 (1) Å	θ = 2.9–28.3°
*c* = 15.6131 (3) Å	µ = 6.58 mm^−^^1^
β = 106.177 (1)°	*T* = 100 K
*V* = 1225.71 (4) Å^3^	Prism, colourless
*Z* = 4	0.20 × 0.11 × 0.07 mm

### 1-Bromo-4-[2-(4-chlorophenyl)ethyl]benzene (II) Data collection

**Table:**

Bruker model? CCD area detector diffractometer	3065 independent reflections
Radiation source: fine-focus sealed tube	2520 reflections with *I* > 2σ(*I*)
Graphite monochromator	*R*_int_ = 0.035
φ and ω scans	θ_max_ = 28.4°, θ_min_ = 2.7°
Absorption correction: multi-scan (SADABS; Sheldrick, 1996)	*h* = −14→14
*T*_min_ = 0.546, *T*_max_ = 0.746	*k* = −10→9
11908 measured reflections	*l* = −20→20

### 1-Bromo-4-[2-(4-chlorophenyl)ethyl]benzene (II) Refinement

**Table:**

Refinement on *F*^2^	Primary atom site location: structure-invariant direct methods
Least-squares matrix: full	Hydrogen site location: inferred from neighbouring sites
*R*[*F*^2^ > 2σ(*F*^2^)] = 0.026	H-atom parameters constrained
*wR*(*F*^2^) = 0.058	*w* = 1/[σ^2^(*F*_o_^2^) + (0.0289*P*)^2^ + 0.0779*P*] where *P* = (*F*_o_^2^ + 2*F*_c_^2^)/3
*S* = 1.03	(Δ/σ)_max_ = 0.001
3065 reflections	Δρ_max_ = 0.45 e Å^−^^3^
145 parameters	Δρ_min_ = −0.40 e Å^−^^3^
0 restraints	

### 1-Bromo-4-[2-(4-chlorophenyl)ethyl]benzene (II) Special details

**Table:**

Geometry. All esds (except the esd in the dihedral angle between two l.s. planes) are estimated using the full covariance matrix. The cell esds are taken into account individually in the estimation of esds in distances, angles and torsion angles; correlations between esds in cell parameters are only used when they are defined by crystal symmetry. An approximate (isotropic) treatment of cell esds is used for estimating esds involving l.s. planes.
Refinement. Owing to poor agreement, the (1 1 1) reflection was omitted from the final cycles of refinement.

### 1-Bromo-4-[2-(4-chlorophenyl)ethyl]benzene (II) Fractional atomic coordinates and isotropic or equivalent isotropic displacement parameters (Å^2^)

**Table:**

	*x*	*y*	*z*	*U*_iso_*/*U*_eq_	
Br1	0.50841 (2)	0.84764 (3)	0.90903 (2)	0.02370 (8)	
Br2	−0.25048 (2)	0.70383 (3)	0.09773 (2)	0.02253 (8)	
C1	0.4150 (2)	0.8055 (3)	0.78827 (15)	0.0159 (5)	
C2	0.3013 (2)	0.7135 (3)	0.77050 (15)	0.0161 (4)	
H2	0.269796	0.672242	0.817887	0.019*	
C3	0.2335 (2)	0.6817 (3)	0.68274 (15)	0.0177 (5)	
H3	0.155194	0.617644	0.670292	0.021*	
C4	0.2778 (2)	0.7417 (3)	0.61232 (15)	0.0167 (5)	
C5	0.3933 (2)	0.8356 (3)	0.63289 (16)	0.0187 (5)	
H5	0.425069	0.878319	0.585874	0.022*	
C6	0.4624 (2)	0.8674 (3)	0.72041 (15)	0.0173 (5)	
H6	0.541066	0.930801	0.733576	0.021*	
C7	0.2051 (2)	0.7034 (4)	0.51691 (16)	0.0255 (6)	
H7A	0.218424	0.577129	0.503799	0.031*	
H7B	0.241041	0.777365	0.477256	0.031*	
C8	0.0625 (2)	0.7386 (3)	0.49531 (15)	0.0212 (5)	
H8A	0.025232	0.652822	0.529264	0.025*	
H8B	0.049949	0.859257	0.516862	0.025*	
C9	−0.0116 (2)	0.7260 (3)	0.39829 (14)	0.0147 (4)	
C10	−0.1344 (2)	0.7992 (3)	0.36922 (15)	0.0167 (5)	
H10	−0.170004	0.854105	0.411688	0.020*	
C11	−0.2056 (2)	0.7945 (3)	0.28122 (15)	0.0172 (5)	
H11	−0.288380	0.846752	0.263034	0.021*	
C12	−0.1540 (2)	0.7119 (3)	0.21965 (15)	0.0161 (4)	
C13	−0.0336 (2)	0.6354 (3)	0.24522 (15)	0.0172 (5)	
H13	0.000460	0.578442	0.202509	0.021*	
C14	0.0369 (2)	0.6430 (3)	0.33433 (15)	0.0154 (4)	
H14	0.119669	0.590830	0.352166	0.018*	

### 1-Bromo-4-[2-(4-chlorophenyl)ethyl]benzene (II) Atomic displacement parameters (Å^2^)

**Table:**

	*U*^11^	*U*^22^	*U*^33^	*U*^12^	*U*^13^	*U*^23^
Br1	0.02109 (13)	0.02960 (15)	0.01712 (13)	−0.00317 (9)	−0.00014 (9)	−0.00484 (9)
Br2	0.01971 (13)	0.03068 (15)	0.01471 (12)	−0.00164 (9)	0.00065 (9)	0.00265 (9)
C1	0.0149 (11)	0.0157 (12)	0.0151 (11)	0.0040 (8)	0.0009 (9)	−0.0027 (8)
C2	0.0160 (11)	0.0163 (11)	0.0175 (11)	0.0019 (8)	0.0073 (9)	0.0018 (9)
C3	0.0136 (11)	0.0205 (12)	0.0185 (12)	−0.0004 (8)	0.0036 (9)	−0.0009 (9)
C4	0.0146 (11)	0.0200 (12)	0.0152 (11)	0.0023 (8)	0.0036 (9)	−0.0006 (9)
C5	0.0146 (11)	0.0216 (12)	0.0214 (12)	0.0015 (9)	0.0076 (9)	0.0038 (9)
C6	0.0106 (10)	0.0172 (12)	0.0243 (12)	−0.0004 (8)	0.0053 (9)	−0.0012 (9)
C7	0.0160 (12)	0.0436 (16)	0.0171 (12)	0.0027 (10)	0.0050 (10)	−0.0026 (11)
C8	0.0166 (12)	0.0281 (13)	0.0172 (12)	0.0018 (10)	0.0021 (9)	−0.0023 (10)
C9	0.0169 (11)	0.0132 (11)	0.0146 (11)	−0.0018 (8)	0.0054 (9)	0.0007 (8)
C10	0.0173 (11)	0.0152 (12)	0.0195 (12)	−0.0002 (8)	0.0082 (9)	0.0003 (9)
C11	0.0129 (10)	0.0172 (12)	0.0211 (12)	0.0006 (8)	0.0039 (9)	0.0026 (9)
C12	0.0160 (11)	0.0175 (12)	0.0133 (10)	−0.0038 (8)	0.0015 (9)	0.0025 (9)
C13	0.0189 (11)	0.0165 (12)	0.0174 (11)	−0.0011 (9)	0.0070 (9)	−0.0003 (9)
C14	0.0129 (11)	0.0160 (11)	0.0176 (11)	−0.0012 (8)	0.0048 (9)	0.0015 (9)

### 1-Bromo-4-[2-(4-chlorophenyl)ethyl]benzene (II) Geometric parameters (Å, º)

**Table:**

Br1—C1	1.902 (2)	C7—H7B	0.9900
Br2—C12	1.901 (2)	C8—C9	1.507 (3)
C1—C2	1.376 (3)	C8—H8A	0.9900
C1—C6	1.382 (3)	C8—H8B	0.9900
C2—C3	1.384 (3)	C9—C10	1.399 (3)
C2—H2	0.9500	C9—C14	1.399 (3)
C3—C4	1.393 (3)	C10—C11	1.377 (3)
C3—H3	0.9500	C10—H10	0.9500
C4—C5	1.398 (3)	C11—C12	1.388 (3)
C4—C7	1.507 (3)	C11—H11	0.9500
C5—C6	1.385 (3)	C12—C13	1.384 (3)
C5—H5	0.9500	C13—C14	1.390 (3)
C6—H6	0.9500	C13—H13	0.9500
C7—C8	1.516 (3)	C14—H14	0.9500
C7—H7A	0.9900		
			
C2—C1—C6	121.4 (2)	C9—C8—C7	116.1 (2)
C2—C1—Br1	119.02 (17)	C9—C8—H8A	108.3
C6—C1—Br1	119.57 (17)	C7—C8—H8A	108.3
C1—C2—C3	119.2 (2)	C9—C8—H8B	108.3
C1—C2—H2	120.4	C7—C8—H8B	108.3
C3—C2—H2	120.4	H8A—C8—H8B	107.4
C2—C3—C4	121.3 (2)	C10—C9—C14	117.4 (2)
C2—C3—H3	119.4	C10—C9—C8	119.73 (19)
C4—C3—H3	119.4	C14—C9—C8	122.9 (2)
C3—C4—C5	117.9 (2)	C11—C10—C9	122.3 (2)
C3—C4—C7	121.1 (2)	C11—C10—H10	118.8
C5—C4—C7	120.9 (2)	C9—C10—H10	118.8
C6—C5—C4	121.3 (2)	C10—C11—C12	118.6 (2)
C6—C5—H5	119.3	C10—C11—H11	120.7
C4—C5—H5	119.3	C12—C11—H11	120.7
C1—C6—C5	118.8 (2)	C13—C12—C11	121.3 (2)
C1—C6—H6	120.6	C13—C12—Br2	119.32 (17)
C5—C6—H6	120.6	C11—C12—Br2	119.41 (17)
C4—C7—C8	114.2 (2)	C12—C13—C14	119.0 (2)
C4—C7—H7A	108.7	C12—C13—H13	120.5
C8—C7—H7A	108.7	C14—C13—H13	120.5
C4—C7—H7B	108.7	C13—C14—C9	121.4 (2)
C8—C7—H7B	108.7	C13—C14—H14	119.3
H7A—C7—H7B	107.6	C9—C14—H14	119.3
			
C6—C1—C2—C3	0.3 (3)	C7—C8—C9—C10	−163.7 (2)
Br1—C1—C2—C3	−179.64 (16)	C7—C8—C9—C14	16.4 (3)
C1—C2—C3—C4	−0.3 (3)	C14—C9—C10—C11	−1.3 (3)
C2—C3—C4—C5	0.0 (3)	C8—C9—C10—C11	178.8 (2)
C2—C3—C4—C7	178.7 (2)	C9—C10—C11—C12	0.9 (3)
C3—C4—C5—C6	0.4 (3)	C10—C11—C12—C13	0.0 (3)
C7—C4—C5—C6	−178.3 (2)	C10—C11—C12—Br2	−179.99 (16)
C2—C1—C6—C5	0.0 (3)	C11—C12—C13—C14	−0.5 (3)
Br1—C1—C6—C5	179.96 (16)	Br2—C12—C13—C14	179.49 (16)
C4—C5—C6—C1	−0.4 (3)	C12—C13—C14—C9	0.1 (3)
C3—C4—C7—C8	46.6 (3)	C10—C9—C14—C13	0.7 (3)
C5—C4—C7—C8	−134.8 (2)	C8—C9—C14—C13	−179.4 (2)
C4—C7—C8—C9	172.1 (2)		

### 1-Bromo-4-[2-(4-chlorophenyl)ethyl]benzene (II) Hydrogen-bond geometry (Å, º)

*Cg*1 and *Cg*2 are the centroids of the (C1–C6) and (C9–C14) rings, respectively.

**Table:**

*D*—H···*A*	*D*—H	H···*A*	*D*···*A*	*D*—H···*A*
C3—H3···*Cg*2^i^	0.95	2.69	3.442 (2)	136
C6—H6···*Cg*1^ii^	0.95	2.91	3.704 (2)	141
C13—H13···*Cg*2^iii^	0.95	2.87	3.569 (2)	131

Symmetry codes: (i) −*x*, −*y*+1, −*z*+1; (ii) −*x*+1, *y*+1/2, −*z*+3/2; (iii) −*x*, *y*−1/2, −*z*+1/2.
